# Supplementary material for: RNF185 destabilizes specific membrane proteins that span all ERAD branches
Source: bioRxiv. 2026 Sep 24:2026.09.23.753758. Preprint. [Version 1] doi: 10.64898/2026.09.23.753758 (PMC13622213; doi:10.64898/2026.09.23.753758)
Supplement: Supplementary file 1 [file NIHPP2026.09.23.753758v1-supplement-4.pdf]

bioRxiv preprint doi: https://doi.org/10.64898/2026.09.23.753758; this version posted September 24, 2026. The copyright holder for this preprint (which was not certified by peer review) is the author/funder, who has granted bioRxiv a license to display the preprint in perpetuity. It is made available under a CC-BY 4.0 International license.

Supplemental Figures

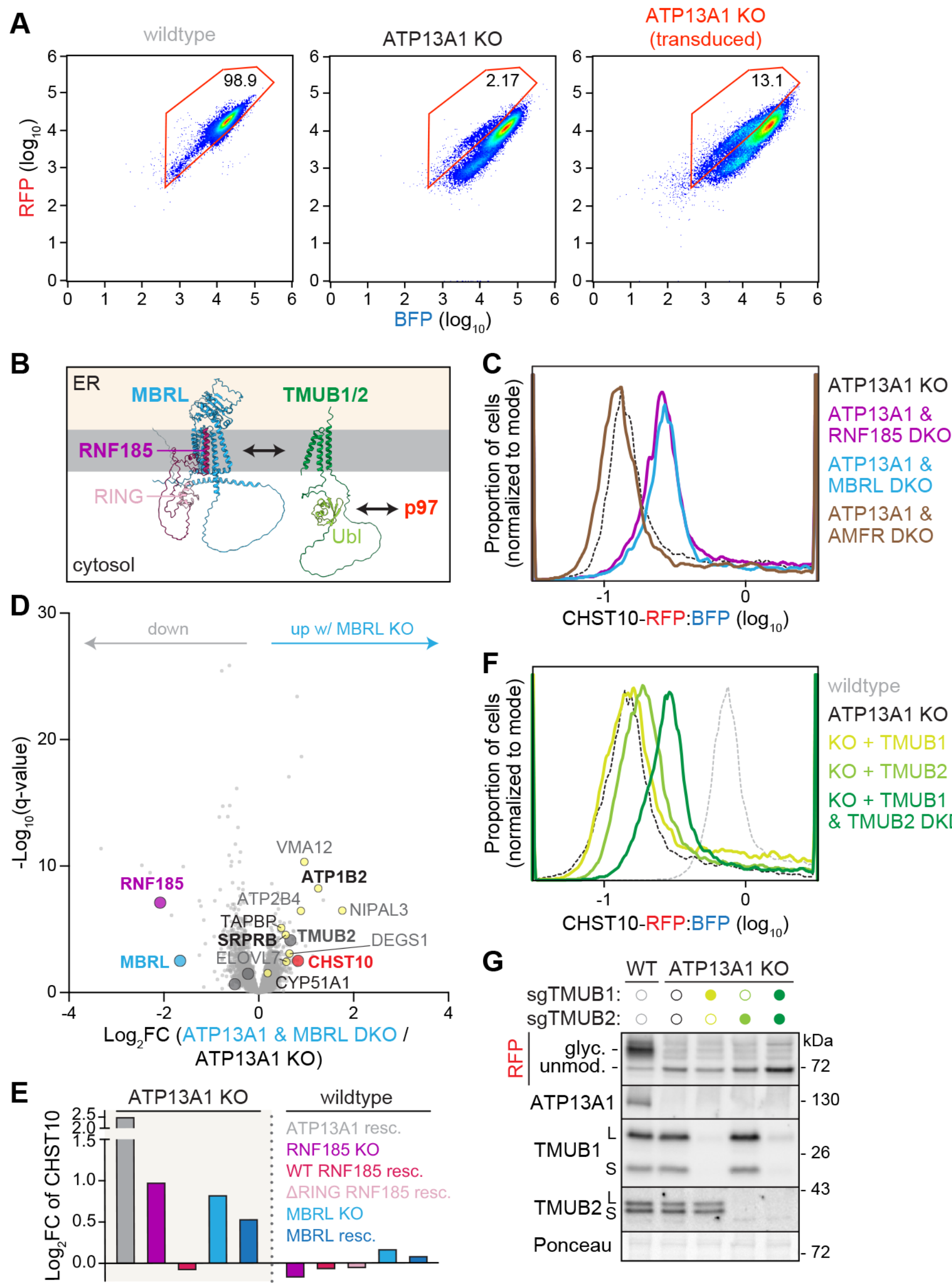

Figure S1. Factors that destabilize misoriented CHST10.

(A) Fluorescent flow cytometry scatter plots of RFP vs. BFP levels in wildtype or ATP13A1 knockout (KO) Flp-In 293 T-REx cells expressing the CHST10 stability reporter without or with transduction (right) of the TKOv3 library. Percentage of cells within the gate is indicated. Note: TKOv3 library transduction increases the proportion of ATP13A1 KO cells with higher RFP:BFP levels.

(B) Alphafold models of RNF185 (purple) in complex with MBRL (blue) and of TMUB1 (green). The RNF185 RING domain (light purple) and TMUB1 Ubl domain (light green) are indicated.

(C) Knocking out RNF185 or MBRL stabilizes CHST10 in ATP13A1 KO cells. Fluorescent flow cytometry of the CHST10 stability reporter in wildtype (gray) or ATP13A1 KO (black) cells

bioRxiv preprint doi: https://doi.org/10.64898/2026.09.23.753758; this version posted September 24, 2026. The copyright holder for this preprint (which was not certified by peer review) is the author/funder, who has granted bioRxiv a license to display the preprint in perpetuity. It is made available under a CC-BY 4.0 International license.

without or with additional knockout (double knockout, DKO) of RNF185 (purple), MBRL (light blue), or AMFR (brown).

(D) TMT-MS volcano plot showing changes in protein levels upon knocking out MBRL (DKO) in ATP13A1 knockout (KO) Flp-In T-REx cells.

(E) Fold-change (Log_2FC) in CHST10 levels relative to ATP13A1 KO (left, shaded) or wildtype (right) cells upon ATP13A1 re-expression (gray), RNF185 KO without (purple) or with re-expression of WT (hot pink) or ΔRING (lavender) RNF185, or MBRL KO without (light blue) or with re-expression (dark blue) of MBRL.

(F) Fluorescent flow cytometry of wildtype (gray) or ATP13A1 KO (black) cells expressing the CHST10 stability reporter without or with polyclonal sgRNA-mediated knockdown (KD) of TMUB1, TMUB2, or both (double knockdown, DKD). Note: knocking down both TMUB1 and TMUB2 stabilizes CHST10 in ATP13A1 KO cells.

(G) SDS-PAGE and immunoblotting of lysates from cells as in (F).

bioRxiv preprint doi: https://doi.org/10.64898/2026.09.23.753758; this version posted September 24, 2026. The copyright holder for this preprint (which was not certified by peer review) is the author/funder, who has granted bioRxiv a license to display the preprint in perpetuity. It is made available under a CC-BY 4.0 International license.

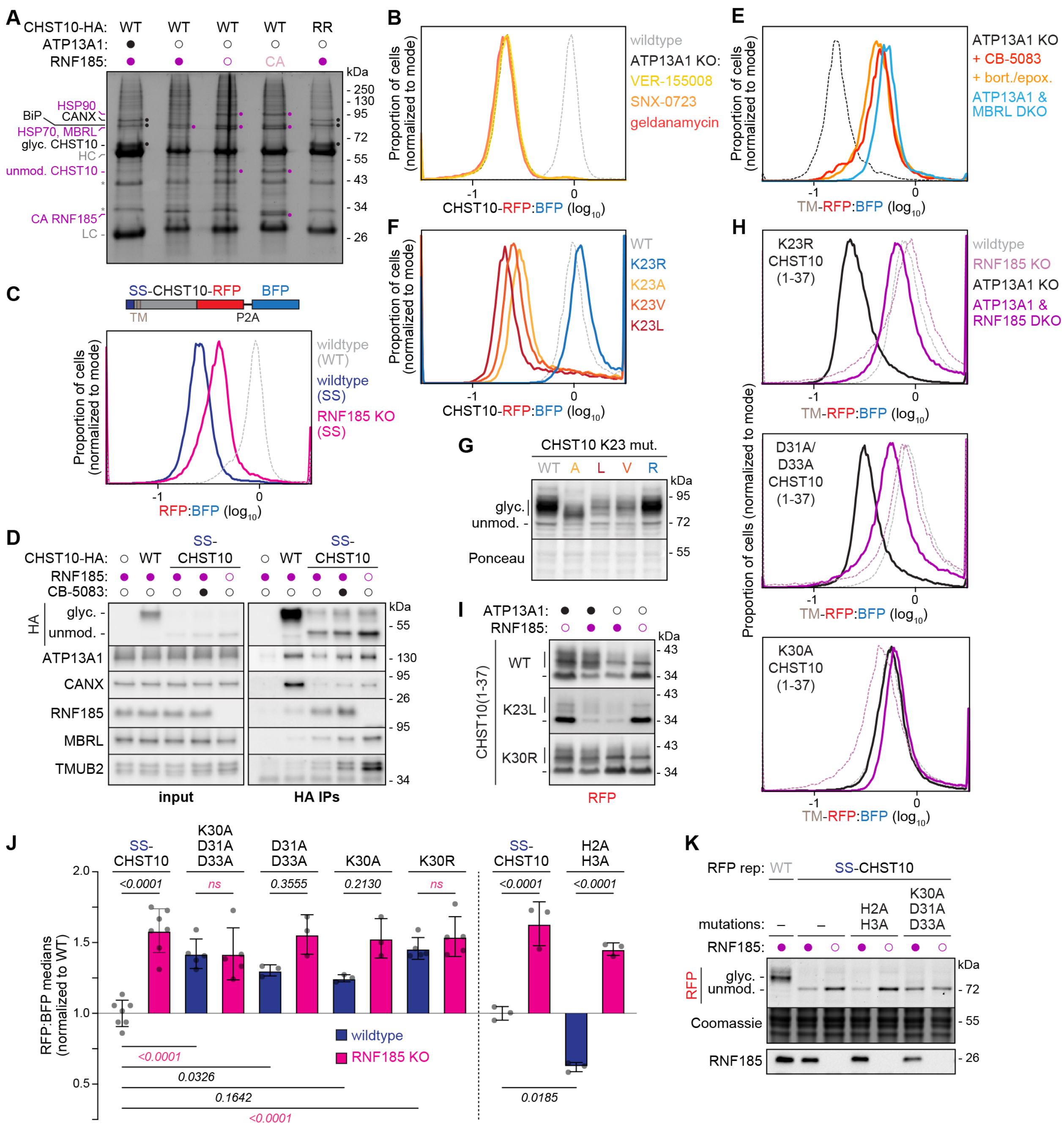

Figure S2. TM misorientation drives CHST10 destabilization.

(A) Misoriented CHST10 specifically interacts with the RNF185 complex. SDS-PAGE and Coomassie staining of affinity purifications of wildtype (WT) or H2R/H3R (RR) C-terminally HA-tagged CHST10 in cells without (filled circles) or with knockout of ATP13A1 (open black circles) and/or RNF185 (open purple circles), and with re-expression of catalytically inactive C76A/C79A (CA) RNF185 (lane 4). Glyc. and unmod. CHST10 and near-stoichiometric proteins, identified by mass spectrometry, that co-elute with correctly oriented (black dots and labels) or misoriented (purple dots and labels) CHST10, are indicated. HC – heavy chain, LC – light chain, * – nonspecific interactors of antibody.

bioRxiv preprint doi: https://doi.org/10.64898/2026.09.23.753758; this version posted September 24, 2026. The copyright holder for this preprint (which was not certified by peer review) is the author/funder, who has granted bioRxiv a license to display the preprint in perpetuity. It is made available under a CC-BY 4.0 International license.

(B) Fluorescent flow cytometry histograms of the RFP:BFP ratios of the CHST10 stability reporter in wildtype (left) or ATP13A1 KO (right) cells without or with treatment for 7 hr with inhibitors against HSP70 (30 μM VER155008) or HSP90 (500 nM SNX-0723, 2 μM geldanamycin).

(C) Scheme (top) of the SS-CHST10-RFP stability reporter with the N-terminal cleavable SS of preprolactin appended to CHST10 and fluorescent flow cytometry (bottom) of wildtype or RNF185 KO Flp-In 293 T-REx cells expressing the wildtype (WT) CHST10 or the SS-CHST10 stability reporter.

(D) SDS-PAGE and immunoblotting of lysates before (bottom) or after anti-HA immunoprecipitations (IPs, top) of C-terminally HA-tagged WT CHST10 or SS-CHST10 in wildtype (filled purple circles) or RNF185 KO (open purple circles) cells treated without or with 1 μM CB-5083 (filled black circle) for 6 hr. Note: SS-CHST10 is not glycosylated and specifically interacts with the RNF185 complex.

(E) Fluorescent flow cytometry of ATP13A1 knockout (KO) without or with additional MBRL KO (blue) Flp-In 293 T-REx cells expressing a stability reporter containing the first 37 amino acids of CHST10 appended to RFP (TM-RFP) and separated from BFP by a P2A ribosome skipping sequence, treated for 6 hr without (black) or with p97 AAA-ATPase (1 μM CB-5083, dark orange) or proteasome (0.5 μM bortezomib, bort., and 0.5 μM epoxomicin, epox., light orange) inhibitors. Note: the CHST10 TM reporter in ATP13A1 KO cells is stabilized by the inhibitors and by MBRL KO.

(F) Fluorescent flow cytometry histograms of the RFP:BFP ratios of the full-length CHST10 stability reporter in wildtype cells without (WT, gray) or with mutation of K23 to arginine (blue), alanine (light orange), valine (orange), or leucine (dark red).

(G) SDS-PAGE and immunoblotting of lysates from cells as in (F). Note: K23A, K23L, and K23V all show reduced levels of glycosylated CHST10. Because K23A displays a different glycosylation pattern, we focused analysis on K23L.

(H) Fluorescent flow cytometry of the RFP:BFP ratios of the minimal CHST10-TM stability reporter with the indicated mutation(s) in the indicated cell lines.

(I) SDS-PAGE and immunoblotting for RFP of lysates from cells without (filled circles) or with (open circles) ATP13A1 and/or RNF185 KO expressing the indicated CHST10-TM reporters. Note: the K23L mutation reduces reporter glycosylation (vertical line) and independently destabilizes the reporter in wildtype cells, while the K30R mutation stabilizes unmodified reporter (dash) populations in all cell lines.

(J) Individual (dots) and average (bars) median RFP:BFP ratios +/- s.d. from >3 independent fluorescent flow cytometry measurements of the indicated SS-CHST10 stability reporter variants in wildtype (navy) or RNF185 KO (pink) cells. P-values (one-way ANOVA) between the values in wildtype and RNF185 KO cells for each variant (top) and between SS-CHST10 and each variant in wildtype cells (bottom) are indicated. Measurements of the H2A/H3A variant were collected with different laser settings and are compared to measurements of the SS-CHST10 reporter collected under the same conditions.

(K) SDS-PAGE and in-gel RFP fluorescence and Coomassie staining (top) or immunoblotting (bottom) of lysates from wildtype (filled circles) or RNF185 KO (open circles) cells expressing WT CHST10 or the indicated SS-CHST10 variant reporter.

bioRxiv preprint doi: https://doi.org/10.64898/2026.09.23.753758; this version posted September 24, 2026. The copyright holder for this preprint (which was not certified by peer review) is the author/funder, who has granted bioRxiv a license to display the preprint in perpetuity. It is made available under a CC-BY 4.0 International license.

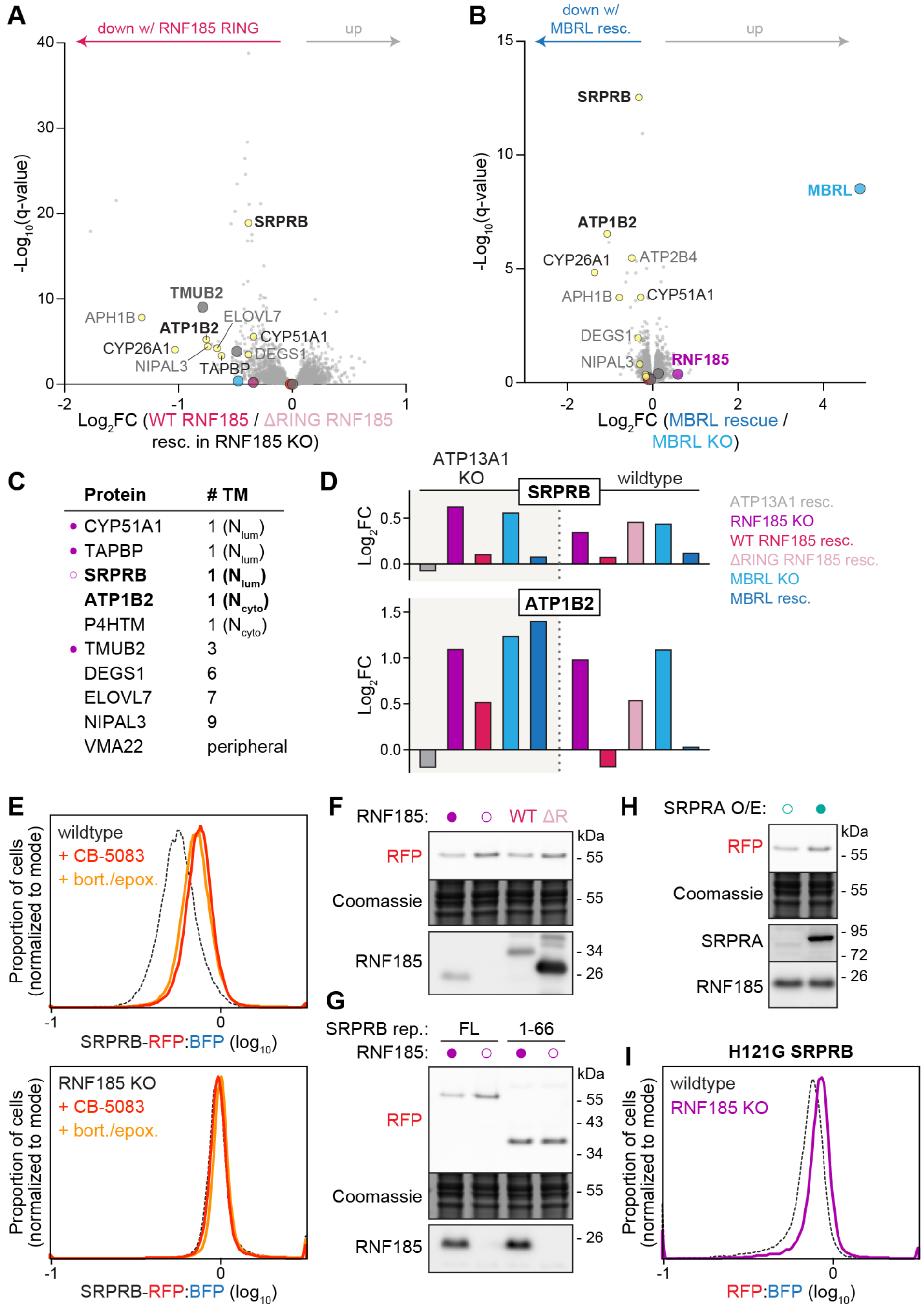

Figure S3. Identification of membrane proteins destabilized by the RNF185 complex.

(A) TMT-MS volcano plot showing differences in protein levels in RNF185 KO cells re-expressing either wildtype (WT) or ΔRING Flag-tagged RNF185. Colored dots indicate RNF185 (purple), MBRL (light blue), CHST10 (red), and other RNF185-sensitive proteins (yellow).

(B) TMT-MS volcano plot showing changes in protein levels upon re-expressing Flag-tagged MBRL (rescue) in MBRL KO Flp-In 293 T-REx cells.

(C) Endogenous proteins identified to be destabilized by RNF185 in both wildtype and ATP13A1 KO backgrounds, with their predicted number of transmembrane helices (# TM) and

bioRxiv preprint doi: https://doi.org/10.64898/2026.09.23.753758; this version posted September 24, 2026. The copyright holder for this preprint (which was not certified by peer review) is the author/funder, who has granted bioRxiv a license to display the preprint in perpetuity. It is made available under a CC-BY 4.0 International license.

topology for single-spanning proteins. Previously noted RNF185 substrates (filled purple circles) and interactors (open circle) are indicated.

(D) Fold-change (Log_2FC) in the levels of SRPRB (top) or ATP1B2 (bottom) relative to ATP13A1 KO (left) or wildtype (right) cells upon ATP13A1 re-expression (resc., gray), RNF185 KO without (purple) or with re-expression of WT (hot pink) or ΔRING (lavender) RNF185, or MBRL KO without (light blue) or with re-expression (dark blue) of MBRL.

(E) Fluorescent flow cytometry histograms of the RFP:BFP ratios of a stability reporter of RFP-tagged SRPRB, separated from BFP by a P2A ribosome skipping sequence, in wildtype (top) or RNF185 knockout (KO, bottom) Flp-In 293 T-REx cells without (black) or with inhibitors of p97 AAA-ATPase (1 µM CB-5083, dark orange) or proteasome (0.5 µM bortezomib, bort., and 0.5 µM epoxomicin, epox., light orange) activity for 6 hr.

(F) SDS-PAGE and in-gel RFP fluorescence and Coomassie staining (top) or immunoblotting (bottom) of the SRPRB stability reporter in wildtype (filled circle) or RNF185 KO cells without (open circle) or with re-expression of WT or ΔRING (ΔR) Flag-tagged RNF185.

(G) As in (F) of stability reporters of full-length (FL) or the first 66 amino acids (1-66) of SRPRB in wildtype (filled circles) or RNF185 KO (open circles) cells.

(H) As in (F) of the SRPRB stability reporter in wildtype cells without (open circle) or with (filled circle) SRPRA overexpression (O/E).

(I) Fluorescent flow cytometry of the H121G SRPRB stability reporter in wildtype (black) or RNF185 KO (purple) cells.

bioRxiv preprint doi: https://doi.org/10.64898/2026.09.23.753758; this version posted September 24, 2026. The copyright holder for this preprint (which was not certified by peer review) is the author/funder, who has granted bioRxiv a license to display the preprint in perpetuity. It is made available under a CC-BY 4.0 International license.

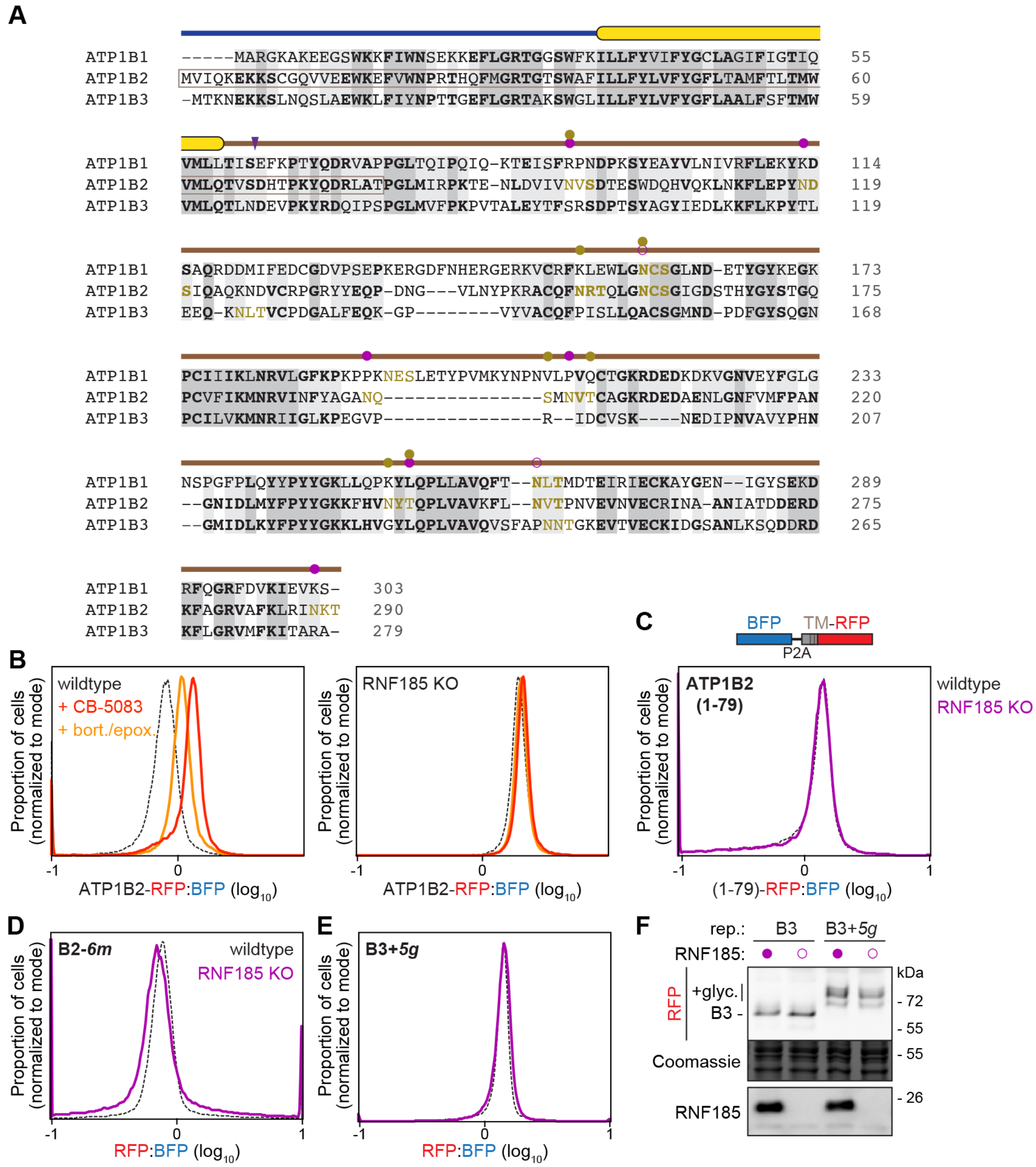

Figure S4. Analysis of ATP1B paralog variants.

(A) Sequence alignment of ATP1B1, ATP1B2, and ATP1B3, with the cytosolic N-terminal domain (dark blue line), predicted transmembrane helix (yellow), and lumenal domain (brown) indicated above. The first 79 amino acids of ATP1B2 (boxed) used for the TM reporter, the chimera switch site (arrowhead), and N-linked glycosylation motifs (gold) are shown. Purple and gold dots denote the locations of loss-of and gain-of glycosylation mutations made in ATP1B2 and ATP1B3, respectively. Open purple dots – mutations only in the ATP1B2-*8m* mutant.

(B) Fluorescent flow cytometry histograms of the RFP:BFP ratios of a stability reporter of RFP-tagged ATP1B2 following BFP and a P2A ribosome skipping sequence in wildtype (left) or RNF185 KO (right) cells without (black) or with inhibitors of p97 AAA-ATPase (1 μM CB-5083, dark orange) or proteasome (0.5 μM bortezomib, bort., and 0.5 μM epoxomicin, epox., light orange) activity for 6 hr.

bioRxiv preprint doi: https://doi.org/10.64898/2026.09.23.753758; this version posted September 24, 2026. The copyright holder for this preprint (which was not certified by peer review) is the author/funder, who has granted bioRxiv a license to display the preprint in perpetuity. It is made available under a CC-BY 4.0 International license.

(C) Scheme (top) and fluorescent flow cytometry histograms of the RFP:BFP ratios (bottom) of a stability reporter of the first 79 amino acids of ATP1B2 (1-79) in wildtype (black) or RNF185 KO (purple) cells. Note: this reporter is not sensitive to RNF185.

(D) Fluorescent flow cytometry histograms of the RFP:BFP ratios of the ATP1B2-*6m* stability reporter in which six N-linked glycosylation motifs in ATP1B2 are mutated.

(E) Fluorescent flow cytometry of the RFP:BFP ratios of the ATP1B3+*5g* stability reporter in which five N-linked glycosylation motifs are added to ATP1B3.

(F) SDS-PAGE and in-gel RFP fluorescence and Coomassie staining (top) or immunoblotting (bottom) of lysates from wildtype (filled circles) or RNF185 KO (open circles) cells expressing the indicated RFP-tagged stability reporters of ATP1B3 without or with (ATP1B3+*5g*) mutations to add five N-linked glycosylation sites.
